# Discrimination of outer membrane proteins with improved performance

**DOI:** 10.1186/1471-2105-9-47

**Published:** 2008-01-24

**Authors:** Changhui Yan, Jing Hu, Yingfeng Wang

**Affiliations:** 1 Department of Computer Science, Utah State University, Logan, UT 84322, USA

## Abstract

**Background:**

Outer membrane proteins (OMPs) perform diverse functional roles in Gram-negative bacteria. Identification of outer membrane proteins is an important task.

**Results:**

This paper presents a method for distinguishing outer membrane proteins (OMPs) from non-OMPs (that is, globular proteins and inner membrane proteins (IMPs)). First, we calculated the average residue compositions of OMPs, globular proteins and IMPs separately using a training set. Then for each protein from the test set, its distances to the three groups were calculated based on residue composition using a weighted Euclidean distance (WED) approach. Proteins from the test set were classified into OMP versus non-OMP classes based on the least distance. The proposed method can distinguish between OMPs and non-OMPs with 91.0% accuracy and 0.639 Matthews correlation coefficient (MCC). We then improved the method by including homologous sequences into the calculation of residue composition and using a feature-selection method to select the single residue and di-peptides that were useful for OMP prediction. The final method achieves an accuracy of 96.8% with 0.859 MCC. In direct comparisons, the proposed method outperforms previously published methods.

**Conclusion:**

The proposed method can identify OMPs with improved performance. It will be very helpful to the discovery of OMPs in a genome scale.

## Background

Outer membrane proteins (OMPs) perform diverse functional roles, including bacterial adhesion, structural integrity of the cell wall, and material transport [1-3]. The membrane-spanning regions of OMPs form a characteristic β-barrel. Discriminating OMPs from other proteins and identifying membrane spanning β-barrels in them are crucial for many studies. Unlike α-helical membrane proteins, which can be easily identified based on long stretches of hydrophobic residues, OMPs are more difficult to predict, mainly due to shorter membrane-spanning regions with higher variations in properties [3]. Nevertheless, several methods have been proposed for this task. Gnanasekaran et al. [4] used profiles developed from structure-based alignments of porins to identify OMPs. Wimley et al. [5] analyzed the structure of 15 non-redundant OMPs and developed a method to identify OMPs based on residue composition and structural features, such as rise-per-residue of the β strands and loop length. Martelli et al. [6], Bagos et al. [7,8], and Bigelow and Rost [9] used hidden Markov models (HMMs) to predict the topology of OMPs and discriminate OMPs from globular proteins. Liu et al. [10] developed a method that combines the residue composition of membrane-spanning regions and predicted secondary structure to identify OMPs. Natt et al. [11] used artificial neural network (ANN) and support vector machine (SVM) methods to identify β-barrels in OMPs using primary sequence, evolutionary information and physicochemical parameters as input. Their method also achieved success in discriminating OMPs. Garrow et al. [12,13] developed a method for discrimination of OMPs in genomes using K-nearest neighbor method. Berven et al. [14] developed the BOMP method that predicts OMPs by combining pattern search, β-barrel score, and a filter that explores the abundance of asparagine and isoleucine in the protein. Gromiha and Suwa [15] developed a simple statistical method to identify OMPs based on amino acid composition. Later, they extended the approach by adding residue pair information and used a SVM-based method to identify OMPs with improved performance [16].

In this study, we propose a simple method that discriminates OMPs from non-OMPs using a weighted Euclidian distance (WED) calculated from residue composition. Our results show that this method achieves 96.8% accuracy with 0.859 MCC. In direct comparisons, the proposed method outperforms previous published methods.

## Results

### Discrimination between OMPs and non-OMPs

For each protein, we calculated its weighted Euclidean distances (WEDs) to OMP, inner membrane protein (IMP) and globular protein groups separately. Then, proteins were classified into OMP versus non-OMP (i.e., IMPs + globular proteins) classes based on the least WED. We explored three different approaches to calculate the WEDs: (I) Only the protein of interest was used to calculated residue composition. Then, the composition of the total 20 amino acids was used to calculate WEDs; (II) Homologous sequences were included in the calculation of residue composition. Then, the composition of the total 20 amino acids was used to calculate WEDs; and (III) Homologous sequences were used to calculated residue composition and a feature-selection method was used to select a set of residues and di-peptides that were useful for OMP prediction. Then, the composition of the selected set was used to calculate WEDs. The results (Table 1, rows 2) show that approach I achieves 91.0% accuracy and 0.639 MCC. Comparisons (Table 1, rows 2–4) show that the classification performance was gradually improved by including homologous information (Approach II) and using feature selection (Approach III). In the end, when approach III is used, the method achieves 96.8% accuracy and 0.859 MCC.

**Table 1:** Performance of the published method and comparisons with previous methods with on-line servers

Mehod		MCC	Accuracy (%)	Sensitivity (%)	Specificity (%)
WED ^a^	Single ^b^	0.639	91.0	77.2	92.9
	Homologous ^c^	0.648	91.4	76.3	93.5
	Homologous + feature selection ^d^	**0.859**	**96.8**	90.7	97.6
BOMP (Berven et al., 2004)		0.817	96.2	79.8	98.5
ProfTMP (Bigelow and Rost, 2006)		0.583	92.3	37.0	1
TMB_HUNT (Garrow et al. 2005)		0.828	96.4	81.5	98.5

a. The method proposed in this study. Proteins were classified based on the least weighted Euclidean distance (WED).

b. For each protein, only the protein itself was used to calculate residue composition.

c. For each protein, 50 homologous proteins were included in the calculation of residue composition.

d. For each protein, 50 homologous proteins were included in the calculation of residue composition. Feature-selection was used to select a set of residues and di-peptides that were useful for the prediction of OMPs. Weighted Euclidean distances were then calculated based on the composition of the selected set.

### Comparisons with previously published methods

We compare the proposed method with previously published methods. As discussed in Baldi et al. [17], in a two-class classification, if the numbers of examples in the two classes are not equal, MCC is a better measure for evaluating the classification performance. In the discrimination of OMPs and non-OMPs, the numbers of examples in the two classes are not equal. Therefore, we will use MCC as the primary measure in the comparison of different methods. At the same time, we also report accuracy, specificity, and sensitivity.

BOMP [14], TMB-Hunt [12,13] and PROFtmb [9] are three top-scoring on-line servers that can discriminate OMPs. BOMP and TMB-Hunt are based on the K-nearest neighbor method, and PROFtmb is based on a hidden Markov model (HMM). We compared the proposed method with these methods by submitting the datasets used in this study to these servers. The comparisons (Table 1, rows 4–7) show that the proposed method outperforms all the other methods. It is worth to point out that the datasets used in this study are likely to have a big overlap with the datasets that were used to train BOMP, TMB-Hunt and PROFtmb servers. Thus, when we evaluated these methods by submitting our datasets to their web servers, the performance of these methods might have been overestimated. Remarkably, our method still outperforms the others under this condition.

Researchers in Suwa's group [15,16,18] developed three methods for discriminating OMPs based on amino acid composition. Here, we compare our method with theirs. In one of their studies, Gromiha and Suwa [15] developed a simple statistical method to discriminate OMPs based on the least "deviation distance", which was calculated as $\sum_{i}\left|{\bar{x}}_{i}-x_{i}\right|$, where *x*_*i *_is the composition of residue type *i *in the test protein, ${\bar{x}}_{i}$ is the average composition of residue type *i *in the target group (OMPs, globular proteins or IMPs). To make direct comparisons, we implemented Gromiha and Suwa's deviation distance method and evaluated it using the datasets used in their study. Then, we repeated our method using their datasets. The comparison (Table 2, rows 2 and 3) shows that our method outperforms Gromiha and Suwa's deviation distance method. In another study, Gromiha and Suwa [19] evaluated a set of 11 machine learning methods for the discrimination of OMPs using residue composition as input. Neural network was reported to achieve the best performance among the 11 methods. Later, researchers from the same group [16] extended the approach by adding residue pair information and used a SVM-based method to identify OMPs with improved performance. In both studies, they evaluated the methods using the same datasets that they used in the "deviation distance" study [15]. We compared the results they reported with the results our method achieved on their datasets. The results (Table 2, rows 2,4,5) show that our method outperforms the neural network methods used in Gromiha and Suwa's study [19]. It is worth to point out that although the same datasets were used to evaluate our method and Suwa's neural network and SVM method, in Suwa's studies, the similarity between training and test sets can be as high as 40%. Meanwhile, we used a stricter criterion to evaluate our method, such that the similarity between training and test set is less than 25%. Even with a stricter criterion used here, our method still outperforms the others.

**Table 2:** Comparisons with other published methods

	MCC	Accuracy (%)	Sensitivity (%)	Specificity (%)
WED (homologous + feature selection)^a^	**0.894**	97.4	91.1	98.4
Deviation Distance [15]	0.541	82.4	78.8	83.3
Neural Network [19]^b^	0.716	91.0	79.3	93.8
Support Vector Machine ^c ^[16]	0.816	93.9	90.9	94.7

a. The method proposed in this study.

b. In their study, Gromiha and Suwa [19] evaluated 11 different methods. Neural network was reported to be the best.

c. The statistics are obtained from the original publications [16, 19]. In the original publications, only Accuracy, Sensitivity and Specificity were reported. Here, we calculate the MCC based on their published statistics.

### Receiver Operating Characteristic (ROC) Curve

**Figure 1:**
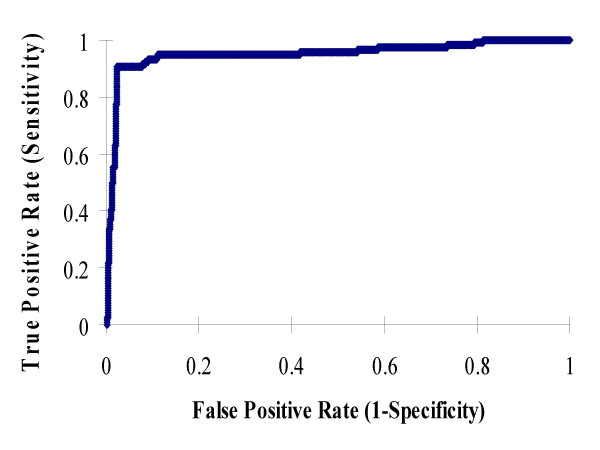
ROC curve of the proposed method.

In the proposed method, a protein is classified as OMP or non-OMP based on the comparison of *D*_*omp *_(its distance to the OMP group), *D*_*glo *_(its distance to the globular protein group), and *D*_*imp *_(its distance to the IMP group). A protein is predicted to be OMP if *D*_*omp *_< Min{*D*_*imp*_, *D*_*glo *_}, where Min { } returns the minimal value of a set. This criteria is equal to evaluating *D*_*omp *_- Min{*D*_*imp*_, *D*_*glo*_} < 0. In general, we can introduce a threshold parameter α, such that a protein is predict to be OMP if *D*_*omp *_- Min{*D*_*imp*_, *D*_*glo*_} < α. Figure 1 shows the ROC curve of the proposed method obtained by varying α. The ROC curve shows how the method can trade off between specificity and sensitivity by changing α. When applying a prediction method to identify OMPs, some researchers may prefer to identify more potential OMPs (high sensitivity) at the cost of relatively low specificity; others may want to identify OMPs with very high specificity at the cost of relatively low sensitivity. The advantage of introducing this parameter α to the proposed method is that users can chose a threshold based on their need. When α is set to a lower value, the method can achieve higher specificity. On other hand, when a high value of α is chosen, the method can achieve higher sensitivity.

### Identification of OMPs in the Proteome of E. coli

We applied the proposed method to search for OMPs in the proteome of E. Coli using α = -0.05, which corresponds to 98% specificity in the ROC curve. The E. Coli proteome consists of 4,319 proteins. 107 of them were predicted to be OMPs. That accounts for 2.5% of the whole proteome. This ratio is consistent with the previous estimation that 2–3% of the genes in Gram-negative bacteria encodes OMPs [2]. Among these 107 proteins, 49 are annotated as OMP proteins in Swiss-Prot [20] or ePSORTdb [21], a database of protein subcellular locations that have been determined by laboratory experiments, and 15 share very high similarities with some OMPs in the training set (with E ≤ 0.0001 in BLAST comparison). Thus, we have very high confidence in believing that these 64 hits are true positives. In addition, 13 proteins are annotated with "Membrane", "Cell membrane" and "Multi-pass membrane protein" in Swiss-Prot. We submitted these proteins to the TMHMM [22], a server for predicting the topology of trasmembrane α-helical proteins, and PSORTb [23], a server for predicting subcellular locations. None of them was predicted to be trasmembrane α-helical proteins (or inner membrane proteins) by both methods. Thus, most of these 15 proteins are very likely OMP proteins. The remaining 30 proteins may suggest new OMP proteins that have not been previously discovered.

We also compare our method's predictions with the proteome scanning results obtained by BOMP [14]. We choose BOMP for comparison because BOMP's predictions for E. Coli proteins are available on its server. In the E. Coli proteome, BOMP predicted 103 OMP proteins. Comparisons show that 59 proteins were predicted to be OMP by both our method and BOMP. 48 proteins were predicted to be OMP by our method but not by BOMP. Among them, 15 proteins are true positives. Additionally, 9 proteins are annotated with "membrane", "Cell membrane" or "multi-pass membrane protein" in SwissProt. 44 proteins were predicted to be TMB proteins by BOMP but not by our method. Among them, 16 are true positives. Additionally, 7 are annotated with "membrane", "Cell membrane" or "multi-pass membrane protein" in SwissProt. This comparison shows that there is a big overlap between the predictions of the proposed method and BOMP. It also shows that each of the two methods can identify some OMP proteins missed by the other. This suggests the possibility of achieving better performance by combining these two methods. Another possible direction to improve the performance is to combine the current method with other methods that predict signal peptides in proteins, since OMP proteins contain a signal peptide that leads them to the outer membrane.

## Discussion

### Simple Methods versus Complicated Methods

It is estimated that 2–3% of the genes in Gram-negative bacteria encodes OMPs [2]. Identifying all OMPs ("OMPome") from bacterial genome is an urgent and challenging task. Compared with other complicated methods, such as k-nearest neighbor method, neural network and SVM, that have been used to identify OMPs, one merit of the proposed method resides in its simplicity and fast speed. The training data set is read only once. The calculation of residue composition and weighted Euclidean distance (WED) can be done with a very fast speed. The method proposed here will be very helpful to the discovery of "OMPome" in a genome scale.

### Euclidean distance versus weighted Euclidean distance

We used a WED (i.e., $\sqrt{\sum_{i}\frac{{\left({\bar{x}}_{i}-x_{i}\right)}^{2}}{{\bar{x}}_{i}}}$) to discriminate OMPs from non-OMPs. Our results show that this method achieves better performance than a published method that discriminate OMPs and non-OMPs based on a deviation distance (i.e., $\sum_{i}\left|{\bar{x}}_{i}-x_{i}\right|$) [15]. In this study, we also tried Euclidean distance (i.e., $\sqrt{\sum_{i}{\left({\bar{x}}_{i}-x_{i}\right)}^{2}}$) instead of WED. But, the performance is not so good as using WED. Compared with Euclidean distance and the deviation distance, WED can better reveal the relation between a protein and a group. Intuitively, for the same amount of difference (i.e., |${\bar{x}}_{i}$ - *x*_*i*_|), when ${\bar{x}}_{i}$ becomes smaller, the difference will become more significant. For example, for the same amount of difference 0.01 (i.e., |${\bar{x}}_{i}$ - *x*_*i*_| = 0.01), if the composition of residue *i *in OMPs is 90% (i.e., ${\bar{x}}_{i}$ = 0.90 and *x*_*i *_= 0.89), then 0.01 does not imply a significant difference between the test protein and OMPs. But, if ${\bar{x}}_{i}$ = 0.001 (then *x*_*i *_= 0.011), then |${\bar{x}}_{i}$ - *x*_*i*_| = 0.01 will suggest a significant difference between the test protein and OMPs.

## Conclusion

In summary, this paper presents a simple method that can discriminate outer membrane proteins (OMPs) from non-OMPs with high performance: 96.8% accuracy and 0.859 MCC. Direct comparisons show that the proposed method outperforms previously published methods. In addition to its high accuracy and MCC, the proposed method is very simple and can be easily applied to genomic data in large scale.

## Methods

### Datasets

We compiled a set of outer membrane proteins (OMPs) that have been experimentally confirmed. It includes 118 proteins that are classified as "Transmembrane beta-barrels" in the SCOP database [24] and 188 proteins from the "β-Barrel porins" subclass in Transport Proteins Database [25]. We removed redundant proteins so that the mutual identity in the dataset was less than 25%. First the proteins were clustered by running BLASTCLUST with parameters "-S 25 -L 0.9 -b F". This step ensured that any two proteins from different clusters shared less than 25% identical residues over 90% coverage of any protein. Then, one protein was chosen from one cluster. Proteins with less than 50 amino acids and proteins that were not from Gram-negative bacterial were also removed. The final dataset consists of 119 OMPs. Globular proteins and α-helical membrane proteins (inner membrane proteins, IMPs) were obtained from a previous study by Park et al. [16]. We filtered the datasets so that the identity between any two proteins is less than 25%. After the filtering, 673 globular proteins, and 178 IMPs were left.

### Residue composition

Residue composition of a protein was calculated using $x_{i}=n_{i}/\sum_{i}n_{i}$, where *n*_*i *_was the number of residues of type *i *in the protein. Average residue composition of OMPs was calculated using ${\bar{x}}_{i\_omp}=n_{i\_omp}/\sum_{i}n_{i\_omp}$, where *n*_*i*_*omp *_was the total number of residues of type *i *in OMPs. The average residue composition of globular proteins was calculated using ${\bar{x}}_{i\_glo}=n_{i\_glo}/\sum_{i}n_{i\_glo}$, where *n*_*i*_*glo *_was the total number of residues of type *i *in globular proteins. The average residue composition of inner membrane proteins was calculated using ${\bar{x}}_{i\_imp}=n_{i\_imp}/\sum_{i}n_{i\_imp}$, where *n*_*i*_*imp *_was the total number of residues of type *i *in globular proteins.

### Weighted Euclidean distance (WED)

For each test protein, its distance to OMPs was calculated using $D_{Omp}=\sqrt{\sum_{i}\frac{{\left({\bar{x}}_{i\_omp}-x_{i}\right)}^{2}}{{\bar{x}}_{i\_omp}}}$, where *x*_*i *_was the composition of residue type *i *in the test protein, ${\bar{x}}_{i\_omp}$ was the average composition of residue type *i *in OMPs. Note that $\sqrt{\sum_{i}{\left({\bar{x}}_{i\_omp}-x_{i}\right)}^{2}}$ gives the Euclidean distance between the OMP group and the test protein. In this study, we weighted each term inside the summation with 1x¯i_omp. Thus, we call *D*_*omp *_a weighted Euclidean distance (WED). The WED between a test protein and globular proteins (*D*_*glo*_) and the WED between a test protein and inner membrane proteins (*D*_*imp*_) were calculated in a similar way.

### Classification

Proteins were classified into the three groups based on the least WED. A test protein was predicted to be an OMP if *D*_*omp *_≤ *D*_*imp *_and *D*_*omp *_≤ *D*_*glo*_; otherwise, it was predicted to be non-OMP.

### Five-fold cross-validations

Five-fold cross-validations were used to evaluate the proposed method. The overall dataset was divided into five subsets. OMPs, globular proteins and IMPs were distributed into the subsets evenly. In each round of experiment, four subsets were used as the training set and the remaining subset was used as the test set. This procedure was repeated five times with each subset being used as test set once. The average performance was reported.

### Including homologous sequences into the calculation of residue composition

For each protein, the BLAST program [26] was used to search for homologous sequences in the NCBI non-redundant database using an E-value of 0.0001. 50 best hits were chosen from the return result. If less than 50 hits were return, then all of the hits were chosen. These proteins plus the query protein were used to calculate the residue composition for the query protein.

### Feature selection

We extended the proposed method by including the composition of di-peptides. We used a feature selection approach to search for residues and di-peptides that are useful for OMP prediction. For the feature selection, we implemented the *Bestfirst *method in the Weka package [27]. The feature selection was conducted with bi-directional search, with a starting set that include the 20 amino acids. In the end, we obtained a set of features that include the compositions of 14 amino acids and 130 di-peptides.

### Performance measures

Let OMPs to be the positive class and non-OMPs be the negative class. Let *TP *be the number of true positives (i.e., the number of OMPs predicted as OMPs); *TN *be the number of true negatives (i.e., the number of negative proteins predicted as negative); *FN *be the number of false negatives (i.e., the number of OMPs incorrectly predicted as negative) and *FP *be the number of false positives (i.e., the number of negative proteins incorrectly predicted as OMPs). Several measures were used to evaluate the method:

$$\begin{array}{l} \begin{array}{ccc} Sensitivity=\frac{TP}{TP+FN} & Specificity=\frac{TN}{TN+FP} & Accuracy=\frac{TP+TN}{TP+FN+TN+FP} \end{array}\\ MCC=\frac{TP\times TN-FP\times FN}{\sqrt{\left(TP+FN)(TP+FP)(TN+FP)(TN+FN\right)}} \end{array}$$

Sensitivity shows the fraction of OMPs that are correctly identified. Specificity shows the fraction of negative proteins that are correctly identified. Accuracy is the total accurate rate of the predictions. MCC (Matthews correlation coefficient) measures the correlation between predictions and actual class labels, which is in the range of [-1, 1], with 1 denoting perfect predictions. In a two-class classification, if the numbers of examples of the two classes are not equal, MCC is a better measure than accuracy [17]. Therefore, in the comparisons of different methods, we focus on the comparison of MCC. In addition to MCC, we also report the performance in other measures.

### Availability

The software and data sets are available online [28].

## Authors' contributions

CY conceived of and designed the study, performed the analysis and drafted the manuscript. JH and YW participated in computation and discussion. All authors read and approved the final manuscript.
